# Neuromyelitis optica with linear enhancement of corpus callosum in brain magnetic resonance imaging with contrast: a case report

**DOI:** 10.1186/s13256-015-0613-9

**Published:** 2015-06-10

**Authors:** Mohammad Ali Sahraian, Abdorreza Naser Moghadasi, Mahsa Owji, Hoda Naghshineh, Alireza Minagar

**Affiliations:** 1 MS Research Center, Neuroscience Institute, Tehran University of Medical Science, Tehran, Iran; 2 Department of Neurology, LSU Health Sciences Center, 1501 Kings Highway, Shreveport, LA 71103 USA

**Keywords:** Callosal enhancement, Magnetic resonance imaging, Neuromyelitis optica

## Abstract

**Introduction:**

Neuromyelitis optica is a demyelinating disease of the central nervous system with various patterns of brain lesions. Corpus callosum may be involved in both multiple sclerosis and neuromyelitis optica. Previous case reports have demonstrated that callosal lesions in neuromyelitis optica are usually large and edematous and have a heterogeneous intensity showing a “marbled pattern” in the acute phase. Their size and intensity may reduce with time or disappear in the chronic stages.

**Case presentation:**

In this report, we describe a case of a 25-year-old Caucasian man with neuromyelitis optica who presented clinically with optic neuritis and myelitis. His brain magnetic resonance imaging demonstrated linear enhancement of the corpus callosum. Brain images with contrast agent added also showed linear ependymal layer enhancement of the lateral ventricles, which has been reported in this disease previously.

**Conclusions:**

Linear enhancement of corpus callosum in magnetic resonance imaging with contrast agent could help in diagnosing neuromyelitis optica and differentiating it from other demyelinating disease, especially multiple sclerosis.

## Introduction

Neuromyelitis optica (NMO) is a severe demyelinating disease of the central nervous system characterized by optic neuritis and transverse myelitis. The etiology is unknown, but anti-aquaporin 4 (AQP4) receptor immunoglobulin G (IgG) antibody plays an important role in the pathogenesis of the disease. Based on 2006 criteria, diagnosis of NMO requires the presence of optic neuritis and myelitis plus the presence of at least two of the following three characteristics [1]: (1) a contiguous spinal cord lesion, visualized by magnetic resonance imaging (MRI), extending over three or more vertebral segments; (2) MRI criteria not satisfying the revised McDonald diagnostic criteria for multiple sclerosis (MS) [2]; and/or (3) NMO-IgG in serum.

Several studies and case series have shown different patterns of brain involvement in NMO, which include those in the periaqueductal, hypothalamic and periventricular regions, as well as extensive lesions in the cerebral white matter. The corpus callosum has been reported to be involved in NMO in a pattern different from that of MS, such as large edematous lesions or involvement of the callosal edge in a linear pattern. Linear enhancement of the ventricular ependyma has been reported previously as having a “pencil-thin” appearance [3, 4]. In this report, we present a case of a patient with NMO with special MRI features of linear enhancement of the corpus callosum and a pencil-thin appearance in the ventricular ependymal layer.

## Case presentation

**Fig. 1:**
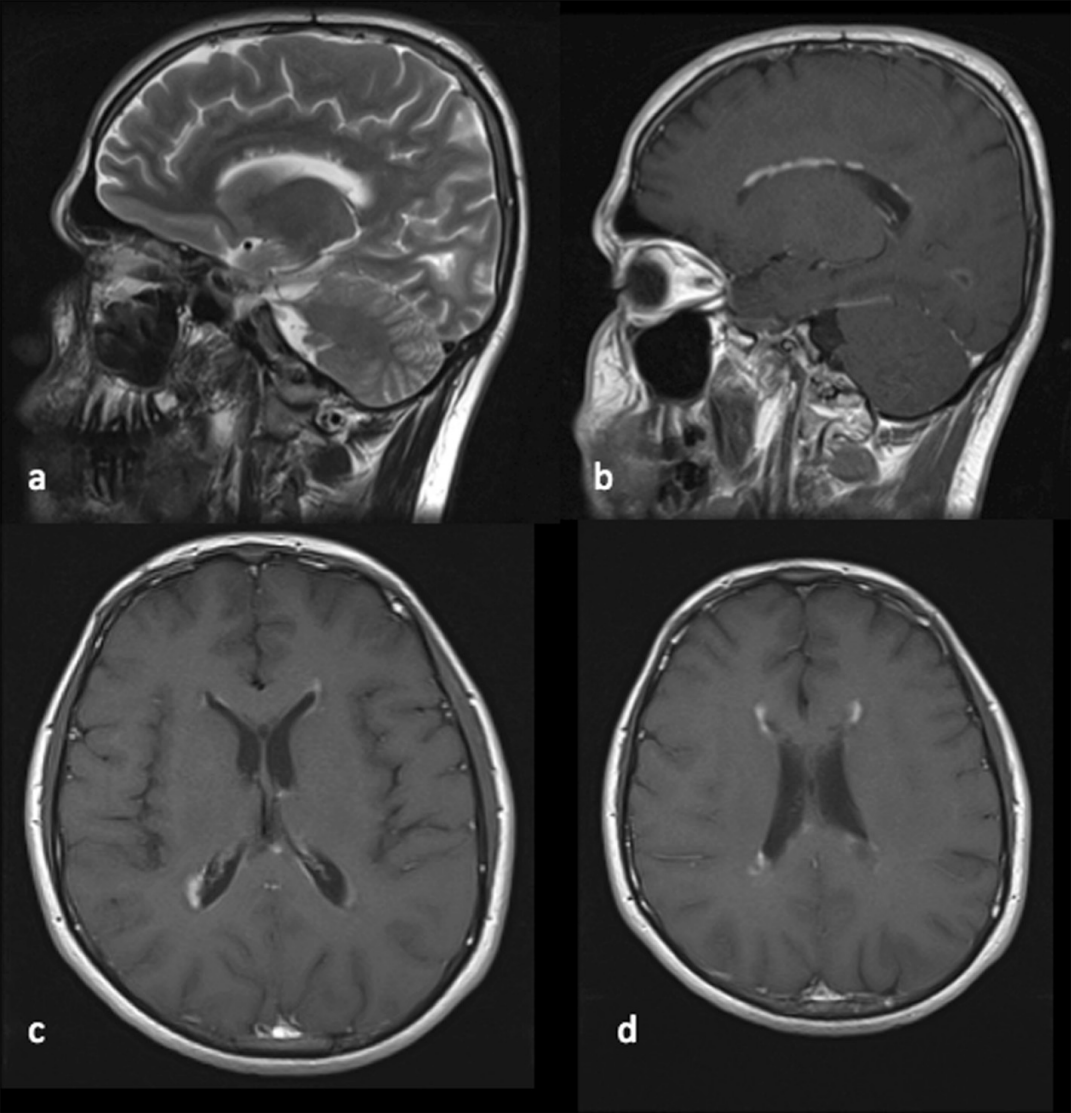
T2-weighted and T1-weighted images with contrast agent. **a**,**b** Images show involvement of the corpus callosum with linear enhancement. **c**,**d** Thin ependymal enhancement of lateral ventricular surfaces (pencil-thin ependymal enhancement)

**Fig. 2:**
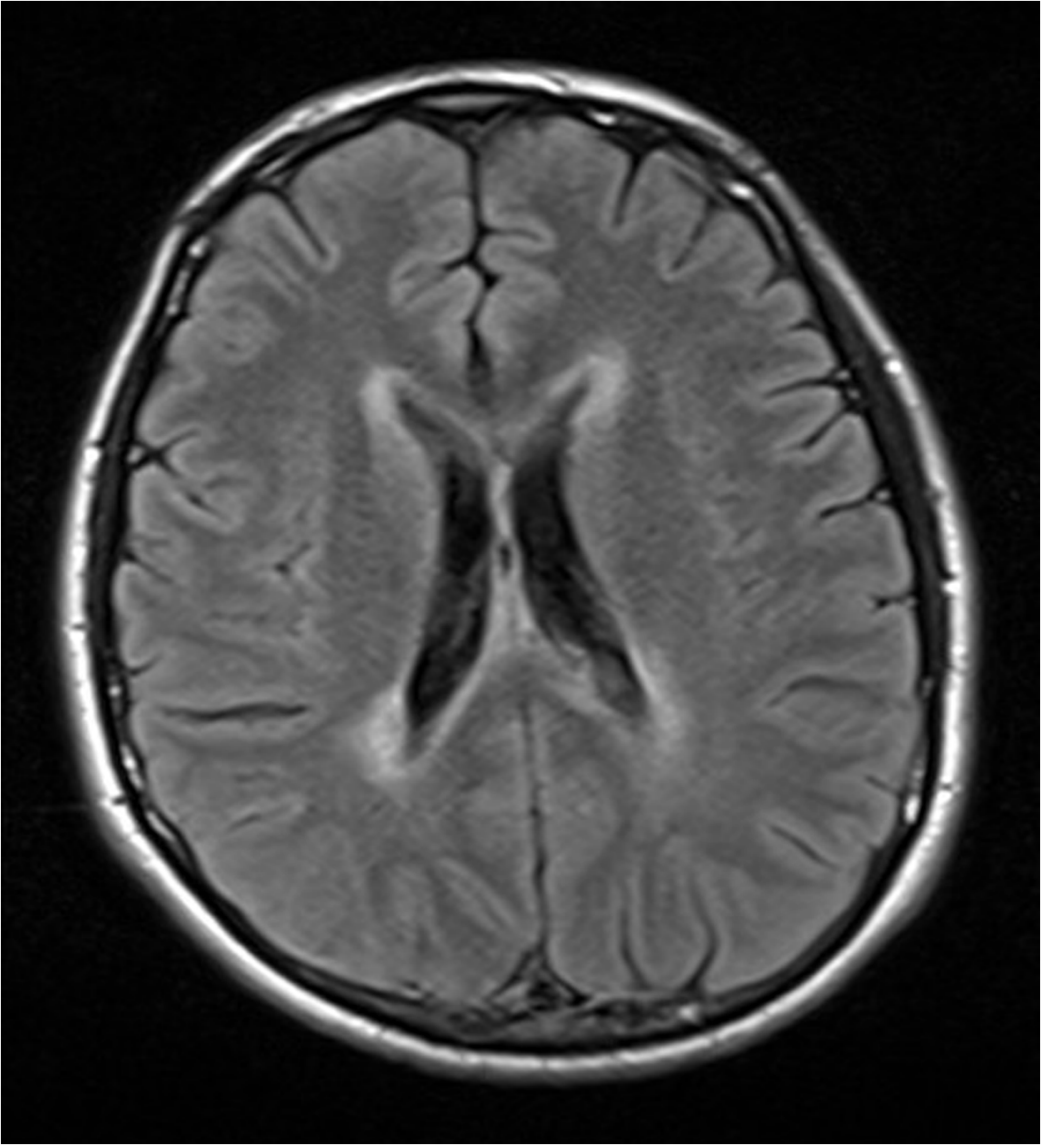
Axial fluid-attenuated inversion recovery magnetic resonance image of the patient shows hyperintense lesions around the ventricles and corpus callosum

Our patient was a 25-year-old Caucasian Iranian man who was referred to our center in 2010 for further evaluation of paraparesis and urinary incontinence. There was no significant point in his past medical or familial medical history. He was well until 2008, when he developed blurred vision in his right eye and was admitted to a local center with the clinical impression of optic neuritis. He received intravenous methylprednisolone 1000mg for 5 days without a good response. Two months later he experienced optic neuritis in his left eye, but he had a good recovery after receiving intravenous methylprednisolone. According to his medical records, his physical examination was normal at that time, with the exception of mild optic atrophy of the right eye. Brain MRI was performed, which revealed some hyperintense T2-weighted lesions around the ventricles, with linear callosal involvement. The lesion was nicely enhanced in a linear pattern in a T1-weighted image with contrast agent (Figs. 1 and 2). Linear enhancement of ventricular surfaces (pencil-thin appearance) in both the frontal and occipital horns was another important finding in this patient.

The patient was diagnosed with MS and received interferon (IFN) beta 1a three times per week. Six months later he developed another episode of optic neuritis in his left eye, and he had a less than 50 % recovery with intravenous steroids and plasmapheresis. After 1 year, he developed paraparesis and urinary incontinence while receiving IFN beta 1a three times per week. He was admitted to a local hospital and received intravenous methylprednisolone 1000mg/day for 5 days. He responded to this therapy and was able to walk in 4 weeks. At that stage, the patient was referred to our center to escalate the disease-modifying therapy.

A neurologic examination of the patient revealed bilateral optic atrophy, weakness of both lower limbs (grade 4/5) and bilateral Babinski sign. Although we did not perform detailed neuropsychological tests, the patient did not show any apparent decrease in cognition or any other signs or symptoms of callosal involvement. New MRI showed a longitudinal cord lesion extending more than four vertebral segments without any enhancement. Regarding episodes of optic neuritis, longitudinal extensive spinal cord lesion and atypical callosal involvement, anti-AQP4 was checked, and the result was positive. Other laboratory tests, such as anti-nuclear antibody and anti-phospholipid antibody, for collagen vascular diseases were negative. The patient was diagnosed with NMO, and azathioprine was started for him at a dose of 50mg/day with a gradual increase to 150mg/day.

## Discussion

NMO is one of the most important mimics of MS. Although NMO has distinct immunopathogenesis and treatment options, both NMO and MS may have similar clinical presentations and imaging features. It is very important to differentiate NMO and MS, especially in patients with both optic nerve and spinal cord involvement, as some MS therapies can exacerbate NMO. MRI may play an important role in differentiating these two entities.

Spinal cord lesions extending over three or more vertebral segments are typical imaging findings in NMO. These longitudinal extensive cord lesions are not specific and can be observed in patients with MS or acute disseminated encephalomyelitis.

Brain MRI scans are usually normal or demonstrate non-specific T2-weighted hyperintense abnormalities. These non-specific small hyperintensities are usually asymptomatic and located in the subcortical or deep white matter area.

Authors of recent case series have reported different patterns of brain involvement in NMO, especially in areas with high AQP4 expression. Diencephalic lesions surrounding the third ventricles and cerebral aqueduct, which include the thalamus, hypothalamus and anterior border of the midbrain, have been reported in NMO. These lesions frequently are asymptomatic, but syndromes of inappropriate anti-diuretic hormone secretion, narcolepsy and hypothermia are some of the clinical manifestations related to these lesions. Lesions located in the dorsal brain stem adjacent to the fourth ventricle (especially area postrema) represent one of the most specific brain MRI abnormalities in patients with NMO and may cause intractable hiccups and vomiting [5].

NMO may involve the corpus callosum in the acute phase with large edematous lesions and heterogeneous intensity, higher in the rim and lower in the core of the lesions (in a “marbled pattern”). Callosal lesions may involve the complete thickness of splenium in a unique pattern that is called an *arch bridge sign* [3, 6]. MS typically and frequently involves the corpus callosum. Lesions in MS are small, discrete, ovoid and perpendicular to the ventricles, and they involve the margin of the corpus callosum. Patients with NMO who have such lesions may be misdiagnosed with MS. This happened to our patient, who was treated with IFN beta 1a for 2 years. It is important for clinicians to know that callosal lesions by themselves are not sufficient to differentiate NMO from MS.

Extensive, confluent, hemispheric, T2-weighted lesions mimicking tumors are more frequent in the pediatric population. These lesions differ from those observed in regions of high AQP4 expression and are likely related to vasogenic edema and sometimes look like “spilled ink” on MRI scans. Such large lesions may follow the white matter tracts without severe mass effect and may cause hemiparesis, encephalopathy and visual field defects [3, 5, 6].

Different patterns of enhancement have been reported to be typical for NMO, such as multiple patchy enhancements with blurred margins in adjacent regions (cloud-like pattern) or linear enhancement of the ependymal surface of the lateral ventricular caps (pencil-thin appearance). Ito and colleagues showed a cloud-like pattern in 90% of their patients with NMO who had contrast-enhanced lesions visualized by MRI [7].

Our patient presented with recurrent optic neuritis and myelitis, which raised the possibility of a diagnosis of NMO or MS. Involvement of the corpus callosum misled the clinician to diagnose the patient with MS and to start MS therapy. Although linear involvement of the corpus callosum and pencil-thin-appearing enhancement of ventricular caps in a patient with optic neuritis could have raised the possibility of NMO, it was ignored at first, and the diagnosis was delayed until the patient developed a longitudinal extensive cord lesion.

To the best of our knowledge, this is the first case report of linear callosal enhancement visualized by MRI. Another interesting neuroimaging feature of this patient is the simultaneous enhancement of the corpus callosum and ventricular surfaces in both the frontal and occipital horns.

## Conclusions

Special types of enhancement previously reported in the literature and the pattern we present in this case report can assist clinicians in differentiating NMO from MS at an early stage and prevent inappropriate treatments. We suggest that clinicians pay special attention to brain MRI of patients who present with optic neuritis and have a poor response to steroids, as well as of patients with extensive cord lesions with atypical brain involvement.

## Consent

Written informed consent was obtained from the patient for publication of this case and any accompanying images. A copy of the written consent is available for review by the Editor-in-Chief of this journal.

## Abbreviations

AQP4: Aquaporin 4
IFN: Interferon
IgG: Immunoglobulin G
MRI: Magnetic resonance imaging
MS: Multiple sclerosis
NMO: Neuromyelitis optica
